# A Preliminary Investigation on a Water- and Acetone-Based Solvolysis Recycling Process for CFRPs

**DOI:** 10.3390/ma17051102

**Published:** 2024-02-28

**Authors:** Christina Vogiantzi, Konstantinos Tserpes

**Affiliations:** Laboratory of Technology & Strength of Materials, Department of Mechanical & Aeronautical Engineering, University of Patras, 26500 Patras, Greece; c.vogiantzi@ac.upatras.gr

**Keywords:** solvolysis, recycling, carbon fibers, mechanical properties, water, acetone

## Abstract

Composites, and especially carbon-fiber-reinforced plastics (CFRPs), are increasingly used in the automotive, aerospace, and aviation industries, and as a result, CFRP production has increased dramatically, leading to a corresponding increase in waste. Landfills and the incineration of waste are likely to be restricted as a result of legislation, thus highlighting the need for efficient recycling methods for CFRPs. However, the recycling of CFRPs is very challenging, mainly due to the difficulty of removing their thermosetting matrix. This study reports a pre-screening of the solvolysis recycling process for CFRPs based on the mechanical properties of the recovered fibers. To this end, solvolysis tests were conducted on unidirectional CFRP samples under supercritical and subcritical conditions using acetone and water. The solvolysis tests were conducted for various conditions of temperature, pressure, and reaction time, without the use of any catalyst. Also, the loading rate (volume of solvent/volume of reactor) was constant. The efficiency of the recycling processes has been evaluated through a morphological and a mechanical characterization of the recovered fibers. In most cases, the decomposition efficiency of the epoxy resin, measured in terms of mass, ranged between 90 and 100%. Moreover, the scanning electron microscopy images of the recovered fibers showed negligible traces of resin residues and no detectable signs of physical damage or any changes in morphology with regard to diameter. Finally, the single-fiber tension tests revealed that that the recovered fibers retained more than 61% of their initial Young’s modulus and 70% of their tensile strength.

## 1. Introduction

Carbon-fiber-reinforced polymers (CFRPs) find extensive applications in the aerospace, aviation, automotive, and energy sectors [1,2]. CFRPs are preferred due to their remarkable specific strength, high modulus, and oxidation resistance, often outperforming traditional, heavier metals. This material choice is associated with expected weight savings, reduced fuel consumption, and environmental benefits. In addition, it is worth noting that FRP (fiber-reinforced polymer) can serve as a corrosion-resistant alternative to steel reinforcement bars, offering further application flexibility [3,4,5]. However, CFRPs currently suffer from resource inefficiency, involving high energy demands during manufacturing and limited recycling options, resulting in a significant portion of composites ending up in landfills [6,7]. The continuous rise in regulations, increasing landfill expenses, and the growing desire for lightweight structures have intensified the need to find methods to recycle composites, and particularly thermoset CFRPs [8,9]. Current recycling methods for CFRPs can be broadly classified into three main categories: mechanical, chemical, and thermal methods. Mechanical recycling involves grinding the CFRP to convert them into particles that can be used again in the manufacturing section. Thermal methods rely on elevated temperatures required for the polymer matrix to undergo vaporization, leaving behind recoverable fibers. Chemical processes use solvents to chemically break down the polymers, preserving the fibers for future use [10]. Mechanical and pyrolysis processes were the primary methods identified for treating composite waste. However, these methods have limitations when it comes to recovering carbon fibers with mechanical properties comparable to those of virgin carbon fibers [11]. Modern recycling methods mainly use pyrolysis, a process that, while recovering carbon fibers as a valuable resource, has disadvantages such as significant energy consumption and the production of brittle fibers that are not suitable for textile applications [12].

In recent years, chemical degradation has emerged as the most promising method for recycling CFRPs, offering the potential to recover residual value from both fibers and chemicals [13]. It is imperative to enhance chemical recycling technology to make it applicable to a wide range of uses in order to become an asset for resource recovery and reduce the environmental impact. Nevertheless, solvolysis has its drawbacks, in particular its dependence on resin types for optimal performance, making the pre-separation of composite types a crucial step [14]. Additionally, the construction of reactors can be costly due to the requirements for durability under high temperatures, pressures, and resistance to corrosion from aggressive solvents. The use of alkaline/acidic solvents and catalysts introduces environmental concerns, leading to the generation of toxic waste. These effluents pose environmental risks, increase disposal costs, and raise potential health concerns. These limitations are important considerations when evaluating the overall feasibility and sustainability of solvolysis processes [15,16]. 

Promising solvents for chemical treatment include water, acetone, and alcohols. In particular, water and alcohols stand out due to their environmental friendliness, wide availability, cost-effectiveness, and low toxicity [17]. For instance, water can be classified as an environmentally friendly reaction medium due to its ready availability, cost-effectiveness, and low potential for toxicity. As a primary solvent, water effectively breaks down the epoxy resin, releasing the virgin fibers under varying decomposition conditions [18]. Also, supercritical acetone offers several advantages, notably its proximity to the Hansen solubility parameter of epoxy resin and its effective solvent capability for epoxy resin degradation [19]. Chemical recycling and in particular solubilization using water and acetone as solvents has been investigated in recent years. Kim et al. [20] investigated solvolysis using water as a solvent (hydrolysis) in supercritical conditions specifically at a temperature of 405 ± 2 °C, pressure of 280 ± 10 bar, and reaction times of 10, 30, 60, and 120 min. They observed that the elimination efficiency of resin was over 99% when the reaction time was 120 min. Yuyan et al. [18] achieved complete decomposition using water in subcritical conditions. Furthermore, the tensile measurements of single fibers showed that the reclaimed fibers exhibited only a 1.8% reduction in tensile strength compared to the virgin fibers. Okajima and Sako [19] investigated the removal of epoxy resin using acetone under superheated and supercritical conditions at 350 °C, with the decomposition efficiency increasing with the reaction time up to 60 min to a maximum of 95.6%. Sokoli et al. [21] investigated the degradation of hybrid fiber composites using near-critical water or supercritical acetone. Notably, under specific conditions, supercritical acetone demonstrated the ability to achieve almost complete resin degradation more specifically at a temperature of 280 °C, pressure of 70 bar, and reaction time of 30 min. Furthermore, carbon fibers were effectively retrieved with no significant loss in tensile strength when either water or acetone were used. Okajima et al. [22] explored the chemical recycling of CFRP using supercritical alcohols or ketones as solvents, with optimal degradation achieved through supercritical acetone at a temperature of 320 °C and reaction time of 20 min, and the recovered carbon fibers maintained their original shape and exhibited minimal tensile strength reduction.

The growth of CFRP production has increased the challenge of efficient recycling, requiring sustainable methods to address environmental problems. Current recycling practices, such as mechanical grinding and pyrolysis, have limitations in recovering carbon fibers with mechanical properties comparable to virgin fibers. Recognizing this gap in achieving integrated recycling, our research focuses on chemical recycling–solvolysis as a promising route. In the present work, CFRP samples were subjected to solvolysis using as a solvent supercritical and subcritical water, as well as supercritical acetone. The main objective of the work was to examine the different solvolysis conditions, including the temperature, the pressure, and the reaction duration, which were applied without the use of catalysts. Maintaining a constant loading rate, defined as the volume of solvent relative to the reactor volume, a controlled and reproducible methodology was established. In addition, the effectiveness of the chemical recycling process was assessed by measuring the efficiency of the epoxy resin removal and characterizing the morphology of the fibers using a Scanning Electron Microscope (SEM). Also, the mechanical behavior of the recovered fibers was evaluated using novel single-fiber tensile tests in order to compare the Young’s modulus and tensile strength with those of virgin fibers. These developments significantly enhance the field of CFRP recycling, thus providing a deeper insight into the solvolysis process and its influence on material recovery.

## 2. Experimental

### 2.1. Materials

The CFRPs were supplied by KVE Composites Group (8 plies at 0 degrees), and the unidirectional carbon prepregs (Sigrapreg C U600-0/SD-E501-33%) were manufactured by SGL Carbon. The resin matrix (E501) is an epoxy, and the resin mass content was found to be 33%. The carbon fibers (SIGRAFIL C T50-4.0/240-E100) had a filament diameter of 7.0 μm. To conduct the solvolysis experiments, the specimens were cut into the dimensions of 30 mm × 50 mm using a milling cutter. The thickness of the specimens was 4 mm.

### 2.2. Solvolysis Tests

**Figure 1:**
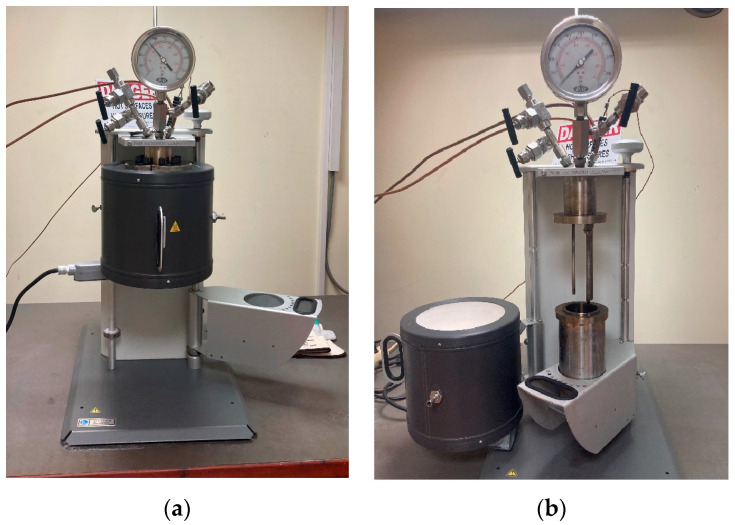
Non-stirred reactor (Parr Instrument Company) (**a**) in operation and (**b**) not in operation.

The epoxy resin in the CFRPs was decomposed using a non-stirred batch reactor (Parr Instrument Company, Moline, IL, USA) shown in Figure 1. The reactor’s vessel is a high-pressure/high-temperature vessel with a 500 mL total volume capacity. The sample was put into the vessel at a loading rate (volume of solvent/reactor volume) of 0.6. The solvolysis tests were conducted using CFRP samples subjected to supercritical and subcritical water and supercritical acetone. The selection of water and acetone as solvents was based on their environmental friendliness, wide availability, cost-effectiveness, and low toxicity, as discussed in the introduction. Five experiments were conducted with a 300 mL solvent. Initially, the system was slowly heated to reach the target temperature. Subsequently, after the predetermined duration (reaction time) of each experiment, the system was cooled down to room temperature through a cooling system (with the use of tap water). In the first series of experiments (#01–03), de-ionized water was used as a solvent under various conditions, and in the last two experiments (#04–05) acetone was used in supercritical conditions. The testing conditions are listed in Table 1. After the cooling, the reactor was unsealed, and the fibers, along with the liquid fraction containing the dissolved organic products resulting from the degraded resin, were collected, and retrieved. Stringent safety precautions, including the use of personal protective equipment (such as gloves and safety glasses) and adequate ventilation, were implemented throughout the experimental procedures. A fume hood cupboard was used for ventilation to actively protect the operator from inhalation of toxic vapors due to resin decomposition. Also, safety and emergency equipment, such as fire extinguishers and a first aid kit, is available in the laboratory.

### 2.3. Characterization of the Recovered Fibers

The resin decomposition efficiency (*RDF*) was measured in terms of mass. The *RDF* is defined as the ratio of the resin mass separated from the fibers to the total mass of the resin before recycling. The *RDF* is calculated using the resin content of the sample, the mass of each sample before the solvolysis experiment, and the mass of the solid residue after recycling [18,23] using

(1) $$RDF=\frac{W_{c}-W_{sr}}{W_{resin}}\times 100\%$$

where $W_{c}$ is the mass of the composite, $W_{sr}$ is the mass of the solid residue, and $W_{resin}$ is the mass of the resin in the composite.

The recycled fiber samples underwent a thorough analysis using a Scanning Electron Microscope (SEM) analysis, specifically in the secondary electron mode. SEM is a well-known technique for the morphological characterization of materials. In SEM, a finely focused electron beam, with energy of 30 kV, scans over a sample surface and the secondary, backscattered electron, etc., are emitted from the surface. The SEM examination was performed using a Zeiss SUPRA 35VP model (Zeiss Group, Oberkochen, Germany), employing a high vacuum mode with a 1.7 nm resolution at a 20 kV accelerating voltage. This analysis aimed to evaluate aspects such as fiber morphology, diameter, and the visual identification of any resin residues.

**Figure 2:**
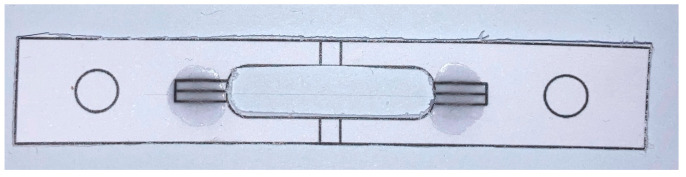
A single-fiber filament bonded to thick paper tabs.

**Figure 3:**
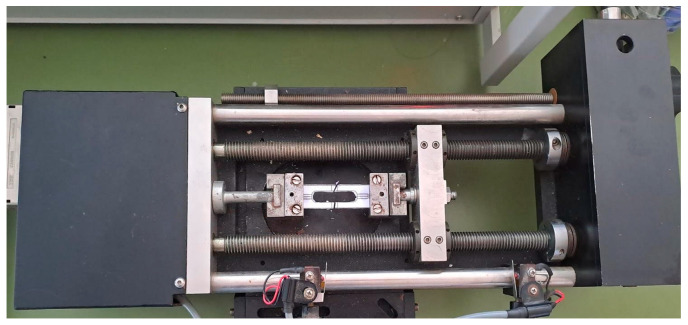
The Miniature Materials Tester Minimat 2000 with a mounted specimen.

Finally, the efficiency of the recycling processes has been evaluated using single-fiber tension tests on the recovered fibers, which were conducted according to the ASTM C 1557-14 standard [24]. From the tests, Young’s modulus and the tensile strength of the recycled fibers were measured. The single-carbon-fiber filaments underwent a meticulous separation process and were temporarily attached to a thick paper mounting tab with a 25 mm gauge length (Figure 2). Once properly positioned and aligned, they were securely bonded using epoxy adhesive. The tests were conducted using a Miniature Materials Tester named Minimat 2000 fitted with a 200 N cell as shown in Figure 3, using a crosshead speed of 2 mm/min. For each sample, a minimum of 25 filaments were tested.

## 3. Results and Discussion

**Figure 4:**
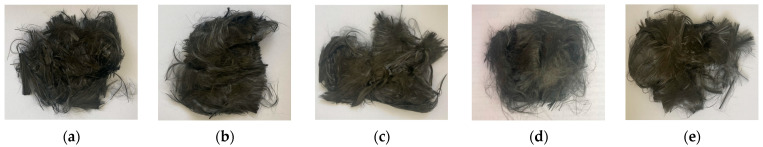
Samples after the solvolysis experiments ((**a**) for the Experiment #01 operating conditions, (**b**) for the Experiment #02 operating conditions, (**c**) for the Experiment #03 operating conditions, (**d**) for the Experiment #04 operating conditions, (**e**) for the Experiment #05 operating conditions).

Following the solvolysis experiments, the recovered fibers were subjected to a treatment process. Specifically, they were immersed in an acetone bath for 20 min to facilitate the removal of any resin residues. After the bath treatment, the humidity was removed by placing the fibers in an oven for 24 h at 40 °C. After the solvolysis process, the samples were visually inspected, and the results are depicted in Figure 4. The visual examination reveals a significant removal of epoxy resin, leaving the fibers in a clean (almost) condition. The length of the fibers appears to be continuous, with negligible changes from their initial length. Additionally, following the solvolysis, the fibers exhibit a non-aligned and fluffy morphology. The recycled carbon fibers typically exhibit this fluffy appearance attributed to the elimination of sizing agents and epoxy resin. 

### 3.1. Resin Decomposition Efficiency

The results from the application of Equation (1) are listed in Table 2. In detail, in Experiment #01, a resin removal efficiency of 91.58% was achieved, demonstrating the effectiveness of the method under specific conditions. Moving on to Experiments #02 and #03, an important Resin Decomposition Factor (*RDF*) was exhibited in both, signifying almost complete elimination of resin, which is particularly noteworthy under supercritical conditions. Experiment #04, however, showed a slightly lower *RDF* of 65.76%, likely due to the lower applied pressure conditions. However, Experiment #05 stood out between the two acetone experiments with an *RDF* of 97.01%, highlighting the consistent performance of the method under high pressure and temperature conditions. In conclusion, in the first three experiments that were conducted using water, the removal efficiency increased with increasing temperature and pressure. The higher rates of resin elimination were achieved under supercritical operating conditions. In Experiment #03, the highest resin removal rate was observed, and it reached almost 100%. However, in Experiment #04, the rate was lower compared to the others. 

### 3.2. SEM Images 

**Figure 5:**
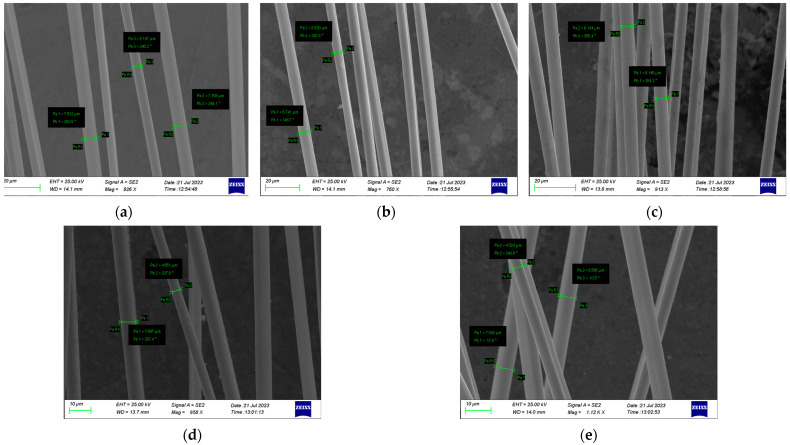
SEM images of recovered fibers ((**a**) for the Experiment #01, (**b**) for the Experiment #02, (**c**) for the Experiment #03, (**d**) for the Experiment #04, (**e**) for the Experiment #05).

The surface microstructures of recovered fibers were observed using SEM. Figure 5 displays the SEM images of them. The recovered fibers showed negligible traces of resin residues on their surfaces, and there were no detectable signs of physical damage such as cracks, or any changes in morphology with regard to diameter. In Experiment #04 (Figure 4d), there is visible resin residue, which confirms the lower rate of resin removal.

Utilizing SEM images, 15 diameter measurements of the fibers for each experiment were also conducted and some of them are displayed in Figure 5. The measured average diameters are listed in Table 3. The values revealed that the mean diameter in every experiment exhibited a negligible change. These negligible changes in diameter could be explained by the removal of the sizing agents during the solvolysis process. Sizing agents are substances applied to fibers during manufacturing to improve adhesion to the matrix. The sizing process, particularly under supercritical conditions where resin removal is greater, can effectively remove sizing agents without causing significant changes in fiber diameter. Therefore, the observation of the diameter in fibers after solvolysis suggests that the method is successful in maintaining the structural integrity of them.

### 3.3. Mechanical Properties and Statistical Analysis

From the single-fiber tension tests on the recovered fibers, the Young’s modulus and the tensile strength were determined and used to assess the recycling process. For the mechanical properties of the virgin fibers, an average value was used, derived from data available in the literature and, more specifically, from [18,25,26,27,28,29,30].

**Figure 6:**
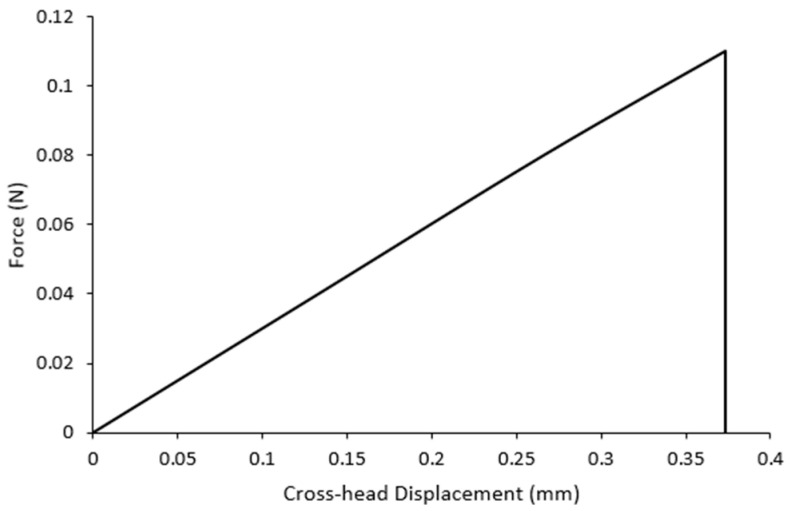
A typical load–displacement curve of a single-fiber tension test.

The constant cross-head displacement rate of the single-fiber tensile test was 2 mm/min, and the maximum possible strength at fracture was obtained within 30 s. A typical load–displacement curve of a single-fiber tension test conducted on a recovered carbon fiber is illustrated in Figure 6. As shown, the fiber exhibited linear tensile behavior.

The maximum load that the fibers could withstand before failure and displacement was recorded for each test, and their average for each experiment was calculated and presented in Table 4. The maximum mean values of the force obtained ranged from 0.065 to 0.075 Newton, while the range of the displacement was 0.34 to 0.4 mm. The displacement provided a quantitative measurement of the ability of the fibers to undergo elongation during the tensile test and will subsequently be used to calculate Young’s modulus.

The Young’s Modulus was calculated based on the ASTM C 1557-14 standard using the following formula:

(2) $$E=\frac{l_{0}}{A\left(\frac{\Delta L}{F_{f}}-C_{s}\right)}$$

where E is the fiber Young’s modulus (Pa), $l_{0}$ is the gage length (m), A is the fiber cross-sectional area at fracture plane (normal to fiber axis) (m^2^), ΔL is the recorded crosshead displacement (m), $F_{f}$ is the force to failure (N), and $C_{s}$ is the system compliance (m/N) which has been measured according to the standard.

**Figure 7:**
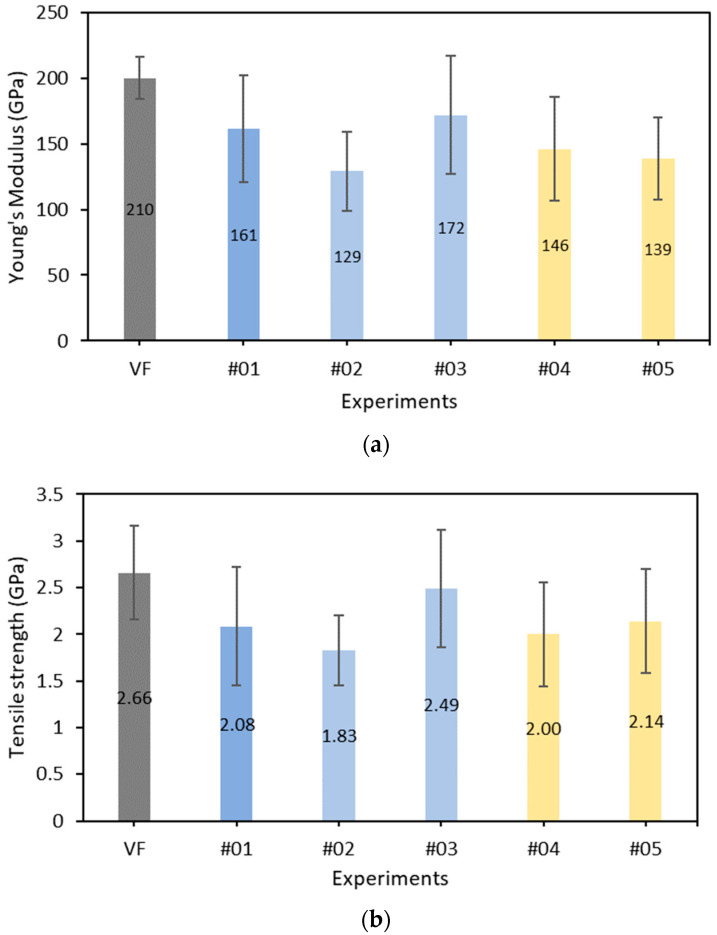
(**a**) The Young’s modulus for the recovered carbon fibers. (**b**) The tensile strength of the recovered carbon fibers.

The derived Young’s modulus values of the different recovered CFs are presented in Figure 7a along with the standard deviation. The literature value for the virgin fibers is 210 GPa. A significant reduction in Young’s modulus of the CFs due to solvolysis varying from 18% for Experiment #03 to 38.6% for Experiment #02 is observed. The reduction varies with the solvolysis conditions. 

As in all experiments, a significant deviation was observed, and to examine the significance of the differences in the Young’s modulus between the recovered and virgin fibers, *t*-tests were conducted. The derived *p*-values from the *t*-tests are 0.151, 0.014, 0.234, 0.081, and 0.012 for Experiments #01 to #05, respectively. Based on α *p*-value of 0.05, Experiments #02 and #05 showed a statistically significant difference, while Experiments #01, #03, and #04 did not. For the latter, this finding suggests that the observed differences may not be statistically significant, indicating a negligible decrease in Young’s modulus.

The tensile strength was derived from the maximum force in the force–displacement curve. The literature value for the virgin fibers is 2.66 GPa. The derived values of tensile strength are presented in Figure 7b along with the standard deviation. Similar to Young’s modulus, a significant reduction of strength is observed ranging from 6.4% to 31%. The extended reaction times during the supercritical solvolysis experiments had a remarkable effect on the mechanical characteristics of the recovered fibers. Again, due to the large standard deviation, *t*-tests were conducted. The resulting *p*-values are 0.054, 0.01, 0.165, 0.026, and 0.045 for Experiments #01 to #05, respectively. Based on a *p*-value of 0.05, it becomes evident that Experiments #02 and #04 yielded statistically significant differences, whereas Experiments #01 and #03 did not. The variation of the tensile strength values of the recovered fibers follows the same trend as Young’s modulus except for the interchange between Experiments #04 and #05. 

## 4. Conclusions

In this paper, a pre-screening of the solvolysis recycling process for CFRPs based on the mechanical properties of the recovered fibers is reported. Solvolysis tests were conducted on unidirectional CFRP samples under supercritical and subcritical conditions using acetone and water. The SEM results reveal that in the majority of experiments, the recycled fibers exhibit a clean (almost), smooth, and continuous morphology, with negligible changes in fiber diameters. Furthermore, the solvolysis process demonstrates excellent decomposition efficiency, ranging between 90% and 100% in four out of five experiments, utilizing only water or acetone as solvents, without the need for any catalyst. Particularly, experiments conducted under supercritical conditions with water showed a direct correlation between increased temperature/pressure and superior resin removal efficiency. Experiment #03, with a shorter reaction time, emerged as excellent, achieving nearly 100% resin removal under water-based solvolysis. A further analysis indicates the importance of optimizing the reaction time, which is particularly evident in Experiment #03. The mechanical tests reveal that the recovered fibers retain over 70% of their initial tensile strength and over 64% of their initial Young’s modulus. The observed variations in tensile strength and Young’s modulus in all experiments underscore the important balance between resin removal and fiber integrity. Supercritical solvolysis experiments have shown that longer reaction times have a remarkable impact on the mechanical properties of the recovered fibers. In Experiment #02 and Experiment #05, extended reaction times led to a significant decrease in tensile strength. However, the important standard deviation observed in the mechanical properties requires careful consideration and indicates the need for further investigation. Statistical tests were initiated to further investigate the variations, revealing significant differences in tensile strength and Young’s modulus between recycled and virgin fibers in specific experiments. These findings not only support the validity of solvolysis as a recycling method but also underline the importance of continuing the research to further improve and optimize CFRP recycling processes. In conclusion, this preliminary study on CFRP recycling using solvolysis has provided important information on the efficiency and potential of this environmentally friendly approach. So, several promising avenues for future research are emerging. The optimization of solvolysis conditions, possible investigation of catalytic effects, and the use of advanced characterization techniques offer opportunities to examine and improve recycling results. Investigating additional methods to examine the mechanical properties of recycled fibers would be beneficial. Also, the application of recycled fibers in CFRPs needs further investigation. Furthermore, continuous monitoring of environmental regulations and standards ensures alignment with evolving sustainability requirements. These future avenues aim to advance the CFRP recycling sector, promoting sustainable practices, and contributing to a circular economy.

## Figures and Tables

**Table 1:** Solvolysis operating conditions for the experiments.

Experiment	Solvent	Temperature (°C)	Pressure (bar)	Reaction Time (min)
#01	Water	320	115	90
#02	Water	380	270	15
#03	Water	400	300	5
#04	Acetone	300	85	60
#05	Acetone	350	230	40

**Table 2:** Input data and *RDF* values.

Experiment	*W_c_* (g)	*W_sr_* (g)	*RDF* (%)
#01	10.72	7.48	91.58
#02	11.45	7.83	95.81
#03	11.05	7.41	99.82
#04	10.23	8.01	65.76
#05	10.62	7.22	97.01

**Table 3:** Diameter measurements.

Carbon Fibers	Average Diameter Values (μm)
Virgin CF	7.00
Experiment #01	6.65
Experiment #02	6.88
Experiment #03	6.19
Experiment #04	6.90
Experiment #05	6.22

**Table 4:** Average maximum force to failure and recorded displacement at failure.

Experiment	Mean Maximum Load (N)	Displacement at Failure (mm)
#01	0.072	0.34
#02	0.068	0.37
#03	0.075	0.38
#04	0.075	0.36
#05	0.065	0.40

## Data Availability

All data are presented in the manuscript.
